# Association between polygenic risk for Alzheimer’s disease and brain structure in children and adults

**DOI:** 10.1186/s13195-023-01256-z

**Published:** 2023-06-13

**Authors:** Xiao-Yu He, Bang-Sheng Wu, Kevin Kuo, Wei Zhang, Qing Ma, Shi-Tong Xiang, Yu-Zhu Li, Zi-yi Wang, Qiang Dong, Jian-Feng Feng, Wei Cheng, Jin-Tai Yu

**Affiliations:** 1 Department of Neurology and National Center for Neurological Disorders, Huashan Hospital, State Key Laboratory of Medical Neurobiology and MOE Frontiers Center for Brain Science, Shanghai Medical College, National Center for Neurological Disorders, Fudan University, Shanghai, China; 2 Institute of Science and Technology for Brain-Inspired Intelligence, Fudan University, Shanghai, China; 3 MOE Frontiers Center for Brain Science, Fudan University, Shanghai, China; 4 Key Laboratory of Computational Neuroscience and Brain-Inspired Intelligence, Ministry of Education, Fudan University, Shanghai, China; 5 ISTBI—ZJNU Algorithm Centre for Brain-Inspired Intelligence, Zhejiang Normal University, Jinhua, China; 6 Zhangjiang Fudan International Innovation Center, Shanghai, China

**Keywords:** Alzheimer’s disease, Polygenic risk score, Genetics, Brain structure, Magnetic resonance imaging

## Abstract

**Background:**

The correlations between genetic risk for Alzheimer’s disease (AD) with comprehensive brain regions at a regional scale are still not well understood. We aim to explore whether these associations vary across different age stages.

**Methods:**

This study used large existing genome-wide association datasets to calculate polygenic risk score (PRS) for AD in two populations from the UK Biobank (*N* ~ 23 000) and Adolescent Brain Cognitive Development Study (*N* ~ 4660) who had multimodal macrostructural and microstructural magnetic resonance imaging (MRI) metrics. We used linear mixed-effect models to assess the strength of the association between AD PRS and multiple MRI metrics of regional brain structures at different stages of life.

**Results:**

Compared to those with lower PRSs, adolescents with higher PRSs had thinner cortex in the caudal anterior cingulate and supramarginal. In the middle-aged and elderly population, AD PRS had correlations with regional structure shrink primarily located in the cingulate, prefrontal cortex, hippocampus, thalamus, amygdala, and striatum, whereas the brain expansion was concentrated near the occipital lobe. Furthermore, both adults and adolescents with higher PRSs exhibited widespread white matter microstructural changes, indicated by decreased fractional anisotropy (FA) or increased mean diffusivity (MD).

**Conclusions:**

In conclusion, our results suggest genetic loading for AD may influence brain structures in a highly dynamic manner, with dramatically different patterns at different ages. This age-specific change is consistent with the classical pattern of brain impairment observed in AD patients.

**Supplementary Information:**

The online version contains supplementary material available at 10.1186/s13195-023-01256-z.

## Background

Dementia, a major global challenge in the twenty-first century, affects approximately 50 million people worldwide and is predicted to reach 152 million by 2050 [1]. As the most common type of dementia, Alzheimer’s disease (AD) is a complex and polygenic disease with a considerable hereditary component (60–80%) [2]. It is a progressive neurodegenerative disorder characterized by concealed onset, and individuals often have significant cognitive impairment and histopathological changes in the brain before overt clinical diagnosis. Given the severe consequences, much attention must be paid to improving risk prediction and facilitating the prevention of the condition.

*APOE ε4* allele is the strongest common genetic risk factor for AD [3], which can elevate the risk of AD and dementia by approximately threefold and advanced age of onset [4, 5]. However, additional common single nucleotide polymorphisms (SNPs) have been discovered by several recent AD genome-wide association studies (GWAS), which may reveal underlying biological mechanistic pathways and offer new perspectives into brain pathology involved in AD-related cognitive decline [6, 7]. Though the individual effect size is minor, a significant modification to AD risk can be achieved when combining these SNPs together. The polygenic risk score (PRS) is developed to quantitatively represent the combined effect of genetic variants on disease risk. It is reported that PRS based on the additive effect of multiple AD-related loci has the potential to work as a valuable predictor of AD risk or pathological trajectories [8, 9].

Magnetic resonance imaging (MRI) markers of brain structure consistently find that the AD-specific symptoms probably result from atrophy and loss of neurons and synapses in particular brain regions [10–12], including the entorhinal cortex, hippocampus, parahippocampal cortex, inferior parietal lobule, cuneus, and precuneus [10, 11]. Moreover, brain white matter shrinkage is also one of the earliest pathophysiological changes detected in AD patients [13]. In general, these results suggest that brain abnormalities might manifest decades before overt clinical symptoms [14–16].

PRS combined with neuroimaging data may provide valuable insights into identifying markers of early risk for AD [17, 18], which will enable strategies for early diagnosis, prevention, and treatment. However, current evidence has mainly focused on the association of AD PRS with global gray and white matter [19, 20] or specific parts such as the hippocampus [21–25], and the investigations linking MRI metrics of comprehensive brain structures at the regional level to genetic variation have so far received much less attention. Furthermore, several studies failed to detect any significant association between PRS and brain measures at a global or regional scale [19, 23]. In addition, only a few studies have investigated these associations in infants [26] or adolescents [27]; the dearth of hard evidence could be due to the undersized number of subjects in earlier genetic neuroimaging studies (*N* < 2000).

In the current study, we analyzed genotype and multimodal MRI data from participants in the UK Biobank (UKB, *N* ~ 23 000) and Adolescent Brain Cognitive Development Study (ABCD, *N* ~ 4660), to assess the associations of genetic loading for AD with multiple MRI metrics of the whole brain. In particular, our study investigated these associations in children and adults by using two cognitively normal populations spanning wide age ranges, as the neural mechanisms during adolescence may vary from those during adulthood. Furthermore, to evaluate the *APOE* impacts on AD PRS, PRS in this study is constructed based on AD-related SNPs, including and excluding the *APOE* region, respectively. We anticipate that the combination of using the existing large GWAS datasets (if applicable) as our training data [6, 7] and using two large populations with MRI imaging data mentioned above as our target data would improve the statistical power to clarify the extent to which these structures evolve at different stages of brain development.

## Methods

### Data source and participants

#### ABCD population

Our investigation used the data from the ABCD consortium annual curated data release 3.0 (https://abcdstudy.org/scientists/data-sharing/), which consists of over 11,878 children aged 9 to 11 years from 21 centers across the USA. Baseline data collection occurred between September 2016 and February 2020 and covered a diverse range of health, socioeconomic, and environmental backgrounds [28, 29]. All parents’ written informed consent and all children’s assent to the research protocol were approved by the Institutional Review Boards at each of the 21 centers [30, 31]. Of the 11,878 participants enrolled, we restricted the sample to European individuals based on self-reported race and genomic ethnic grouping using principal component analysis.

#### UKB population

We also used data from the UK Biobank (www.ukbiobank.ac.uk), a large cohort of 502,486 British males and females (aged 40–69 years at baseline) from 22 assessment centers [32]. UKB obtained ethical approval from the National Health Service National Research Ethics Service, and all participants gave informed consent through electronic signatures. Of all 502,486 participants, 40,077 had MRI data and 488,377 had DNA genotypes, and only participants who were classified as European ethnicity (defined as “white British” using self-reported race and genetic ancestry and confirmed using principal components analysis) [33] with complete DNA genotype information and MRI data were included. Prior to analysis, participants with a diagnosis of dementia (ICD-10 codes: F00, F01, F02, F03, G30, and field 42,018) were excluded.

### Genotyping, data quality control, and imputation

#### ABCD population

The Rutgers University Cell and DNA Repository performed the genotyping of ABCD saliva samples on the Smokescreen array [34], which contains 733,293 SNPs. The following standard preprocessing steps were applied to the genome-wide data [35]. We excluded participants with high rates of missingness (> 5%, *n* = 204), heterozygosity deviating from mean ± 3SD (*n* = 229), and high genetic relatedness (PiHat > 0.2, *n* = 1723). Furthermore, SNPs with call rates < 95%, minor allele frequency (MAF) < 1%, and which were not in Hardy–Weinberg equilibrium (*p* ≤ 1 × 10^−6^) were screened out. Then, imputed data based on the Haplotype Reference Consortium (HRC) panel (v1.1 2016) [36] was conducted through Michigan Imputation Server with Eagle v2.4 phasing, yielding 7,624,970 SNPs in imputation data for 4660 samples.

#### UKB population

Imputed genome-wide genotype data were available for 488,377 individuals in UKB, with details of DNA acquisition, sample manipulation, and quality control (QC) described previously [37]. Two related arrays, including the UK BiLEVE Axiom Array (9.9% participants) and the UK Biobank Axiom Array (about 90% participants) from Affymetrix, were used for genotyping. In addition to sample QC by the UKB team [37], we further removed individuals who have gender-mismatched genetic data (*n* = 625), high missingness rates of > 5% or heterozygosity rate outliers (*n* = 968), and those who are related (have more than ten putative third-degree relatives, *n* = 187), to minimize the potential effects of population stratification. Moreover, SNPs with call rates < 95%, MAF < 0.1%, and departure from the Hardy–Weinberg equilibrium (*p* < 1 × 10^−10^) were excluded, retaining 8,446,385 SNPs available for PRS generation.

In the final analysis, the ABCD sample included 4660 adolescents, comprising 47% females and 53% males aged 9–11 years. The UKB sample included about 23,000 healthy participants, comprising ~ 52% female and ~ 48% male participants with a mean age of ~ 65 years. More details are available in Additional file 2: Table S1.

### Polygenic risk scores

To calculate the PRS, we used PRSice 2 [38], a software package implementing the *p*-value clumping and thresholding method automatically. SNPs were clumped and the most significant associated SNP with *r*^2^ > 0.1 per 250-kb linkage disequilibrium block were retained. After summing the product of the number of SNPs by the weight of each SNP, we generated PRSs for participants in the ABCD and UKB cohorts described above, with the weights being effect sizes derived from the base GWAS summary statistics reported by Schwartzentruber et al. [6] and Kunkle et al. [7], respectively. The former (for ABCD) has a larger sample size, with participants from both the International Genomics of Alzheimer’s Project (IGAP) and the UKB, which is more powerful for assessing genetic liability and plays a more important role in AD risk prediction and stratification. The latter AD GWAS meta-analysis (for UKB) involves only IGAP samples, hence avoiding sample overlap between the base and target data [39]. Eight different *p*-value thresholds (PT, i.e., *p* ≤ 5e − 8, 1e − 6, 5e − 6, 1e − 5, 5e − 5, 0.0001, 0.001, 0.01) were chosen for SNP selection in the construction of PRS to balance signal to noise ratio [40]. The number of SNPs for PRS generation at each *p*-value inclusion threshold can be seen in Additional file 2: Table S2.

### Brain structure

We extracted preprocessed brain imaging data provided by ABCD and UKB teams respectively with all MRI data acquisition and processing pipelines described elsewhere [28, 29, 41], also in Additional file 1: Supplemental Methods 1–3. Regional cortical area (CA), cortical thickness (CT), and cortical volume (CV) of 34 cortical regions defined in the Desikan-Killiany cortical atlas [42]; volume of 7 bilateral subcortical segmentations (SV); fractional anisotropy (FA) and mean diffusivity (MD) of 10 white matter tracks across the left and right hemispheres were used in the following analyses.

Please see a comprehensive list of all MRI metrics examined in our study in Table S3.

### Statistical analysis

**Fig. 1:**
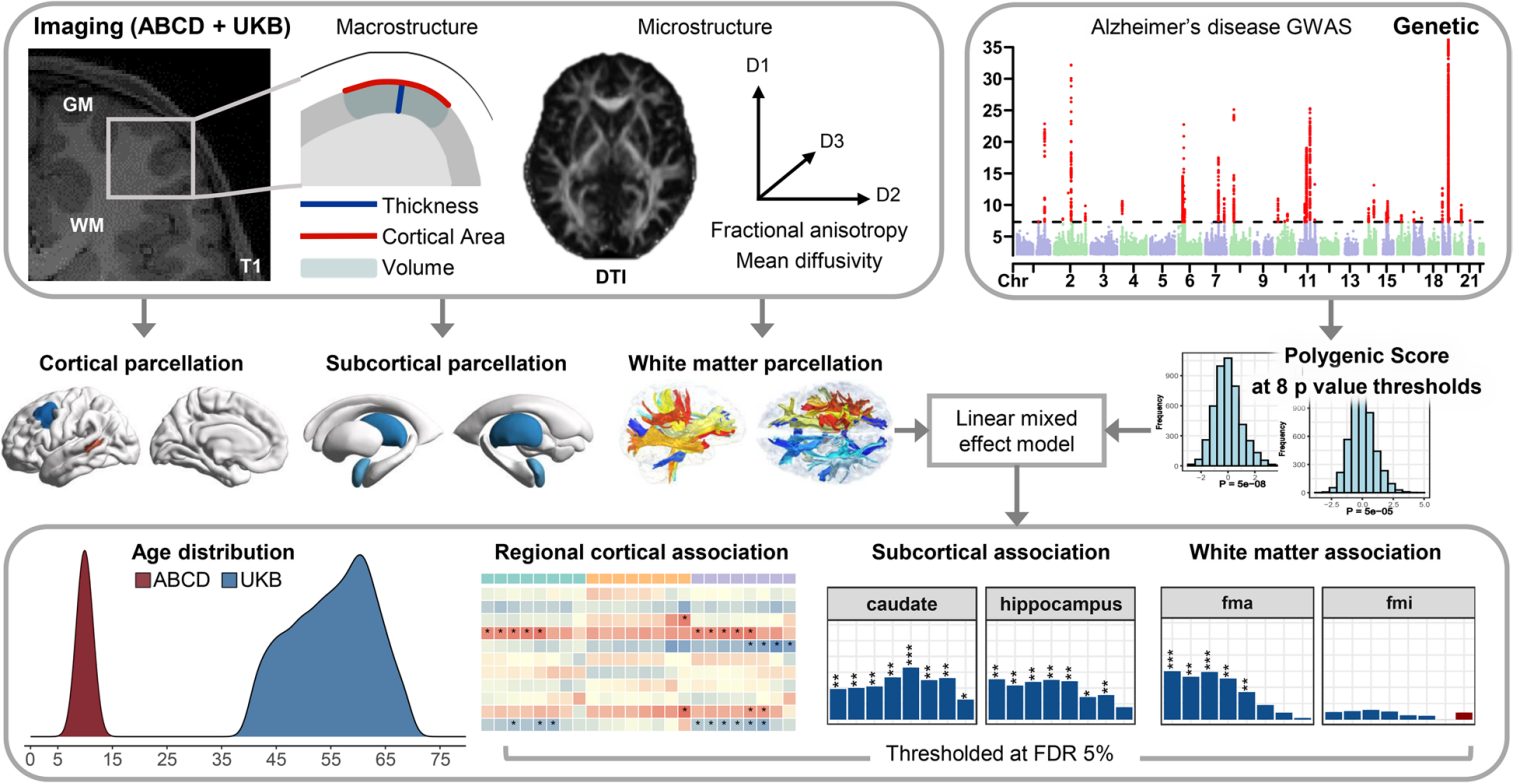
Schematic overview of the study design. We extracted preprocessed brain imaging data provided by ABCD and UKB teams, which includes three macrostructural metrics (CA, cortical area; CT, cortical thickness; CV, cortical volume) at each of the 34 bilateral cortical regions, two microstructural metrics (FA, fractional anisotropy; MD, mean diffusivity) at each of the 10 major white matter tracts, and volume at each of seven subcortical structures (SV, subcortical volume). Polygenic risk scores (PRSs) for Alzheimer’s disease (AD) were calculated for each participant using the clumping and thresholding method at eight *p*-value thresholds for SNP selection. For ABCD and UKB populations, PRSs were based on GWAS data reported previously [6, 7]. We used linear mixed effect models to estimate the association of each brain structure phenotype with each of eight AD PRS in children and adults (age distribution: 9–11 in ABCD, 46–82 in UKB), controlled for multiple comparisons at false discovery rate = 5%. The Manhattan plot is based on a prior published AD GWAS summary data [6] downloaded from www.ebi.ac.uk/gwas/downloads/summary-statistics (under accession GCST90012877) and for illustrative use only. *Abbreviations*: GM, gray matter; WM, white matter; GWAS, genome-wide association study; ABCD, Adolescent Brain Cognitive Development Study; UKB, UK Biobank

The schematic summary of the study is presented in Fig. 1. For bilateral brain regions, linear mixed effect models in the lme4 package [43] in R were used to estimate the association of each scaled brain structure phenotype with the scaled PRS, modeling hemisphere as a random effect after testing for PRS-hemisphere interactions proved to be nonsignificant at false discovery rate (FDR, Benjamini and Hochberg method) = 5% (see Additional file 2: Tables S4-S6) [44, 45]. For unilateral structures (i.e., forceps major and forceps minor), general linear models were used. Nuisance covariates of no interest, i.e., age, age^2^, sex, and the first 10 ancestry principal components (PC), were adjusted as fixed effects in all analyses to correct for subtle population structure effects. We further adjusted for corresponding global metrics separately in the regional cortical analyses and the whole brain volume in the regional subcortical volume analyses, respectively.

$$\mathrm{MRI}\;\mathrm{Metric}\sim\mathrm{PRS}\;+\;\mathrm{age}\;+\;\mathrm{age}^2\;+\;\mathrm{sex}\;+\;10\mathrm{PCs}\;+\;\mathrm{global}\;\mathrm{metric}\left(\mathrm{cortical}\right)\;\mathrm{or}\;\mathrm{whole}\;\mathrm{brain}\;\mathrm{volume}\;(\mathrm{subcortical}),$$

$$\mathrm{random}=\;\sim1\vert\mathrm{Hemisphere}$$

**Fig. 2:**
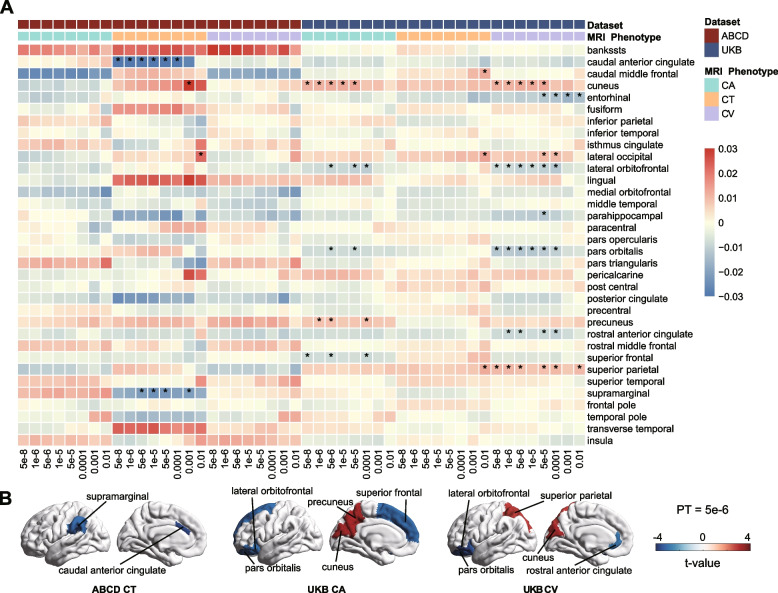
Associations between polygenic risk scores for Alzheimer’s disease and cortical macrostructural MRI metrics. **A** Heatmap of regional associations between CA, CT, CV, and all eight AD PRS. The rows represent the 34 cortical regions, and the columns represent the eight AD PRSs used in our study. Asterisks indicate *p*-values after FDR correction: *FDR < 0.05, **FDR < 0.01. **B** Cortical *t*-maps representing *t*-values of association between AD PRS (at *p*-value threshold = 5e − 6) and regional cortical MRI metrics. Regions where the association with PRS is significant after FDR correction (FDR < 5%) are labeled by name. *Abbreviations*: ABCD, Adolescent Brain Cognitive Development Study; UKB, UK Biobank; CT, cortical thickness; CV, cortical volume; CA, cortical area

**Fig. 3:**
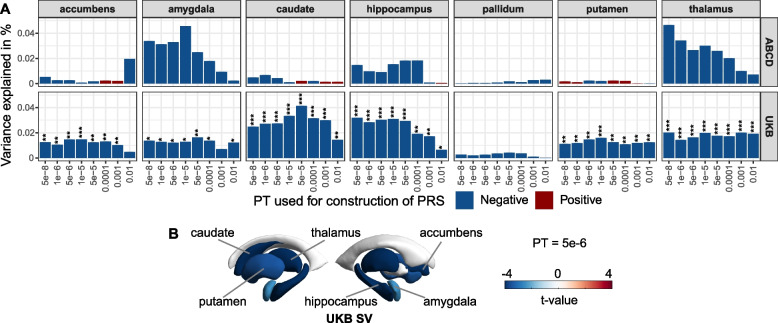
Associations between polygenic risk scores for Alzheimer’s disease and volume of subcortical structures. **A** Barcharts of variance explained by AD PRS (*R*^2^, *y*-axis) constructed at each of eight *p*-value thresholds (*x*-axis) for a volume of 7 subcortical structures: accumbens, amygdala, thalamus, hippocampus, pallidum, caudate, and putamen. Red and blue colors respectively correspond to positive and negative associations. Asterisks indicate *p*-values after FDR correction: *FDR < 0.05, **FDR < 0.01, ***FDR < 0.001. **B** Subcortical *t*-maps representing* t*-values of association between AD PRS (at *p*-value threshold = 5e − 6) and volume of subcortical structures. Regions where the association with PRS is significant after FDR correction (FDR < 5%) are labeled by name. *Abbreviations*: ABCD, Adolescent Brain Cognitive Development Study; UKB, UK Biobank; PT, *p*-value thresholds; PRS, polygenic risk scores; SV, subcortical volume

**Fig. 4:**
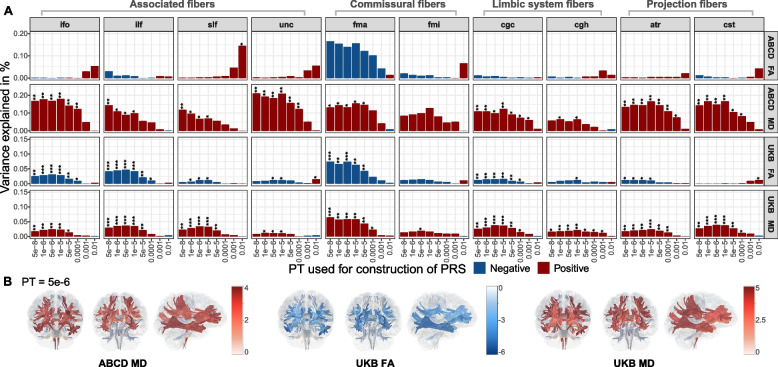
Associations between polygenic risk scores for Alzheimer’s disease and white matter microstructural MRI metrics. **A** Barcharts of variance explained by AD PRS (*R*^2^, *y*-axis) constructed at each of eight *p*-value thresholds (*x*-axis) for two white matter metrics (FA, fractional anisotropy; MD, mean diffusivity) measured at 10 major white matter tracts: ifo, inferior fronto-occipital fasciculus; ilf, inferior longitudinal fasciculus; slf, superior longitudinal fasciculus; unc, uncinate fasciculus; fma, forceps major; fmi, forceps minor; cgc, cingulate gyrus part of cingulum; cgh, parahippocampal part of cingulum; atr, anterior thalamic radiation; cst, corticospinal tract. Red and blue colors respectively correspond to positive and negative associations. Asterisks indicate *p*-values after FDR correction: *FDR < 0.05, **FDR < 0.01, ***FDR < 0.001. **B** White matter *t*-maps representing *t*-values of association between AD PRS (at *p*-value threshold = 5e − 6) and main white matter tracts. Tracts where the association with PRS is significant after FDR correction are shown (FDR value < 5%). *Abbreviations*: ABCD, Adolescent Brain Cognitive Development Study; UKB, UK Biobank; FA, fractional anisotropy; MD, mean diffusivity; PT, *p*-value thresholds; PRS, polygenic risk scores

To avoid the effects of extreme values of MRI metric data, outliers located outside of ± 3 standard deviation (SD) from the mean were removed in each regression. For analysis of 34 cortical regions, 10 white matter tracts, and 7 subcortical structures, we estimated associations with all eight PRSs (Additional file 2: Tables S7-S9), with FDR = 5% within each metric to correct for multiple testing (272, 80, and 56, respectively). Furthermore, we used the PRS at PT 5e − 6, which showed the strongest effect in our analysis [46], to generate *t*-maps of PRS association with cortex, subcortex, and white matter tracts in Figs. 2, 3, and 4.

Furthermore, the following sensitivity analyses were performed. First, to make the PRS in the two cohorts more comparable, we reconstructed PRS in ABCD using the same base GWAS data as for UKB (reported by Kunkle et al., Additional file 1: Figs. S2-S4, Additional file 2: Tables S10-S12). Second, we reanalyzed the MRI metrics with all eight PRSs excluding SNPs within the *APOE* locus (chromosome 19: 44.4–46.5 Mb) [47] along with *APOE* as one of the covariates, to assess whether the association was purely attributable to *APOE* (Additional file 1: Figs. S6-S8, Additional file 2: Tables S13-S15). Third, we included 40 PCs, in accordance with previous literature [48], as covariates in the analysis to capture potential confounding by population stratification (Additional file 2: Table S16-S18). Fourth, we furtherly added the birth weight and gestational age as covariates in the analysis of PRS with the volume of cortical and subcortical structures in ABCD, as brain volumetric analysis in adolescents was often confounded by birth characteristics (Additional file 2: Tables S19-S21). Finally, to maintain data variability and avoid introducing bias, we winsorized the outlier values on MRI metrics to mean ± 3SD deviations rather than removing them (Additional file 2: Tables S22-S24).

## Results

### Regional cortical MRI metrics

In the ABCD samples, CT was significantly negatively associated with PRS in caudal anterior cingulate (maximum *R*^2^ = 0.113%, *β* =  − 0.035; Fig. 2A, Additional file 2: Table S7) and supramarginal (maximum *R*^2^ = 0.053%, *β* =  − 0.024) at more than one PT (FDR = 5%), while none of the regions showed significant associations in cortical CA and CV.

Among the UKB population, CA was significantly associated with PRS in 5 cortical regions at PT = 5e − 6 after correction for multiple testing (Fig. 2B, Additional file 2: Table S7), of which 3 were negative (maximum *R*^2^ = 0.008%, *β* =  − 0.009; superior frontal, FDR = 0.045; pars orbitalis, FDR = 0.045; lateral orbitofrontal; FDR = 0.046) and the remaining 2 were positive (maximum *R*^2^ = 0.014%, *β* = 0.012; cuneus, FDR = 0.037; precuneus, FDR = 0.045). CV had significant associations with PRS in 5 regions at PT = 5e − 6, with 3 negative (maximum *R*^2^ = 0.011%, *β* =  − 0.013; pars orbitalis, FDR = 0.012; lateral orbitofrontal, FDR = 0.020; rostral anterior cingulate, FDR = 0.043) and 2 positive (maximum *R*^2^ = 0.015%, *β* = 0.012; superior parietal, FDR = 0.043; cuneus, FDR = 0.031). We also observed significant associations of CA and CV of cortical regions with AD PRS at other PTs, with a consistent direction of effect sizes on those two MRI phenotypes in similar cortical spatial localization (see the full results in Fig. 2A). According to these results, inverse correlations of AD PRS with CA and CV were concentrated in the prefrontal lobe and cingulate, while the positive relationships were primarily localized in the occipital lobe.

When the subsequent analysis of regional cortical MRI metrics removed the *APOE* locus from PRS, most previously mentioned cortical regions did not report significant correlations (Additional file 1: Fig. S6, Additional file 2: Table S13).

### Regional subcortical MRI metrics

Among ABCD samples, AD PRS calculated at PT = 5e − 6 was nominally associated with reduced SV in the amygdala (Additional file 2: Table S8; *R*^2^ = 0.033%, *β* =  − 0.020, *p* = 0.034) and thalamus (*R*^2^ = 0.027%, *β* =  − 0.018, *p* = 0.019) but not statistically significant after correction for multiple testing (Fig. 3A, Additional file 2: Table S8).

In the UKB population, significant associations of AD PRS with SV at PT = 5e − 6 were found, including the hippocampus (Fig. 3B, Additional file 2: Table S8; *R*^2^ = 0. 030%, *β* =  − 0.018, FDR = 4.98E-05), caudate (*R*^2^ = 0.027%, *β* =  − 0.017, FDR = 3.40E − 05), thalamus (*R*^2^ = 0.016%, *β* =  − 0.013, FDR = 7.22E − 05), accumbens (*R*^2^ = 0.015%, *β* =  − 0.013, FDR = 0.002), putamen (*R*^2^ = 0.015%, *β* =  − 0.012, FDR = 0.001), and amygdala (*R*^2^ = 0.012%, *β* =  − 0.011, FDR = 0.016). These negative associations between PRS and SV conserved significant at more than 4 PTs when correcting for multiple comparisons throughout the seven subcortical structures of interest (Fig. 3A).

Taken together, we found that compared to adolescents, the associations with SV were skewed towards negative values in middle-aged and elderly participants (Fig. 3A), which indicates severe subcortical shrink.

Sensitivity analysis showed most of these associations with AD PRS may be attributable to *APOE* since they were no longer significant after the removal of the *APOE* effect (Additional file 1: Fig. S7, Additional file 2^2^: Table S14).

### Regional white matter MRI metrics

In the ABCD samples, we assessed the association of PRS and two microstructural parameters (FA, MD). There were significant positive associations between AD PRS and MD of nine white matter tracts at a minimum of 2 PTs (Fig. 4A, Additional file 2: Table S9). These effects persisted after the *APOE* locus was removed from the PRS (Additional file 1: Fig. S8, Additional file 2: Table S15).

As for the UKB population, significant positive associations of PRS with MD and negative associations with FA existed for most tracts (Fig. 4A), suggesting that elevated genetic risk for AD was associated with decreased FA, and increased MD in association fibers, commissural fibers, limbic system fibers, and projection fibers. The highest percentage of phenotypic variance explained by AD PRS in association with FA at PT = 5e − 6 was within the commissural fibers (forceps major, *R*^2^ = 0.074%, *β* =  − 0.027, FDR = 5.67E − 04), followed by association fibers (inferior longitudinal fasciculus, *R*^2^ = 0. 047%, *β* =  − 0.022, FDR = 2.38E − 05). The strongest positive correlations between PRS and MD at PT = 5e − 6 were also within commissural fibers (forceps major, *R*^2^ = 0.059%, *β* = 0.024, FDR = 0.001), followed by projection fibers (corticospinal tract, *R*^2^ = 0.039%, *β* = 0.020, FDR = 2.96E − 04) and limbic system fibers (cingulate cingulum, *R*^2^ = 0. 038%, *β* = 0.020, FDR = 2.96E − 04). However, we found almost no significant association after the *APOE* locus was excluded along with adjusted, suggesting that the association was most likely owing to the effect of the *APOE* locus (Additional file 1: Fig. S8, Additional file 2: Table S15).

### Sensitivity analysis

In sensitivity analyses, we wonder whether the age-related changes in the brain structure are attributable to different GWAS used for PRS construction. Pearson correlation analysis was used to calculate the pairwise correlation between different PRS generated by two GWAS summary data in ABCD, and high correlations were reported (5 of 8 PTs had a correlation coefficient above 0.8, with all *p*-value < 2.2E − 16, Additional file 1: Fig. S5A). Furthermore, the results (effect sizes) indicate a high degree of consistency (5 of six brain metrics had a correlation coefficient above 0.7, all *p*-value < 2.2E − 10, Additional file 1: Fig. S5B), though were not significant enough due to the decreased sample size of base GWAS summary data (Additional file 1: Fig. S5C). Moreover, the brain regions which significantly associated with AD-PRS in the main analysis were also significantly associated with AD-PRS generated using GWAS reported by Kunkle et al. although they did not survive after multiple testing correction. Therefore, we suppose this modification in ABCD could not radically alter our results, except to provide more robust effects.

Notably, the main associations did not change substantially after including 40 PCs in UKB (Additional file 2: Tables S16-S18, Additional file 1: Fig. S9) or birth weight and gestational age in ABCD (Additional file 2: Tables S19-S21, Additional file 1: Fig. S10). Moreover, the results with outliers winsorized were highly correlated with results that simply remove outliers (Additional file 2: Tables S22-S24, Additional file 1: Fig. S11), indicating that there were no serious concerns for bias in our study.

## Discussion

Using two different populations with age spans of 9–11 years and 46–82 years (total *N* ~ 27,660), this study examined the strength of the association between AD PRS and different MRI metrics including morphometric and histological measures of comprehensive brain structures in children and adults. This research showed that in adulthood, PRS of AD had associations with regional structure reduction in the rostral anterior cingulate, pars orbitalis, lateral orbitofrontal, superior frontal cortex, hippocampus, thalamus, amygdala, and striatum (caudate, putamen, accumbens), while the brain expansion was concentrated near the occipital lobe (superior parietal, cuneus, and precuneus). Compared to those with lower PRSs, adolescents with higher PRSs had thinner cortex in the caudal anterior cingulate and supramarginal. Additionally, individuals with higher PRSs had widespread microstructural abnormalities in both adolescents and adults, indicated by decreased FA or increased MD in extensive white matter tracts.

The combination of two completely different age-spanning populations enabled us to identify the highly distinct pattern of brain changes and the influence of the *APOE* genotype between adolescents and middle adulthood. This suggested that AD-related brain abnormalities may be partly accounted for genomic vulnerability decades before significant manifestation of clinical symptoms, and caution is needed when assessing the impact across age spans.

### Genetic associations across macrostructural MRI phenotypes of cortex and subcortex

In the cortex, multiple metrics associations with PRS were concentrated in the cingulate and prefrontal lobe, which have previously been reported to show significant cortical atrophy in studies of AD [10, 49–53]. However, the small increased macrostructural measurements in the occipital regions were unexpected. Even though it was also observed by a prior Mendelian randomization study suggesting a causal relationship between AD and greater volume of the occipital lobe [50], the mainstream studies have provided insight into the accelerated rate of occipital lobe atrophy in patients with AD. As massive amyloid deposits can be found in AD patients’ occipital cortex [54], this could be due to its space-occupying effects. It may also be a manifestation of structural brain improvement, as the brain is an active combatant to resist impairment, which may mechanistically result from compensatory neurogenesis or the plasticity in axonal sprouting [55]. Before strong conclusions about different cortical atrophy patterns of AD can be drawn, further analysis would be needed to replicate this finding.

In the subcortex, the results of our exploratory analysis in adolescents did not withstand correction for multiple testing. When taking the lack of significance into account, our analyses either showed that the impact of these risk variants on subcortical structures in children was not significant enough to be detected or it could be attributed to the smaller sample size of ABCD and insufficient statistical test efficacy. For middle-aged and older adults in UKB, the reduced hippocampal volume explains the most variance, a result consistent with the consensus of the hippocampus being the primary focus of neural loss in AD patients [10]. Moreover, the volumetric decline of the amygdala, thalamus, and striatum (caudate, putamen) has also been observed to strongly correlate with PRS, in keeping with previous observational studies of AD demonstrating abnormal shape change [10, 56–58]. In general, our finding reflects the neurodegenerative effect of genetic risk for AD in subcortical areas before clinical manifestations in adults [19, 24, 25].

### Genetic risk and white matter tracts

We demonstrate that in both adolescents and adults, individuals with higher PRSs have increased MD or decreased FA of widespread white matter. The changes in these two metrics possibly represent the loss of neurons, dendrites, and axons in neurodegenerative illnesses like AD, since they reflect less restricted movement of water molecules around the axons’ longitudinal axis and elevated diffusivity of water in all directions [59]. The non-significant results of PRS and FA in adolescents may be due to less susceptibility to white matter structural damage in the younger population. We postulated that adolescents with high genetic risk for AD have a density or activity reduction of terminal neuronal fields in preferentially affected brain regions. These underlying processes would provide a developmental foothold for subsequent pathogenic changes [26], and MD is potentially a more sensitive biomarker to detect compared to FA [59, 60].

Abnormal microstructure of extensive white matter tracts, especially superior and inferior longitudinal fasciculus, cingulate cingulum, corticospinal tract, anterior thalamic radiation, and uncinate fasciculus, has been frequently reported in AD research [23, 61, 62]. These alterations can impact complex networks relevant to episodic memory and other cognitive processes, which have been proven to be associated with immune response genes within AD genetic risk [20]. One possible mechanism is the gene regulatory activation of microglia to alter myelination and axonal growth in immunosurveillance and immune activation manner during development and adulthood [20].

### Comparison of association pattern between AD risk gene and brain in children and adults

There have been inconsistent patterns of association between accelerated biological and brain aging in two populations with different age distributions. Specifically, genetically predicted neurodegenerative results in adults are more proximate to clinical brain pathophysiological changes in AD patients. The alterations associated with genetic risk for AD in adolescents aged 9–11 years appeared to be primarily located in the white matter and cingulate cortex when compared with the middle-aged and older adults and has not yet had a significant impact on subcortical structures. This suggests that specific genetic effects are not strongly exhibited during early neurodevelopment, which may be explained by the temporal and spatial abundance of gene expression.

Genetically mediated decreased CV occurs in different areas within the cingulate in both early and later life, with the former concentrated in the dorsal anterior cingulate and the latter in the rostral anterior cingulate. These two adjacent structures play distinct roles in conflict processing, task monitoring, emotional self-control, social cognitive, and executive functions [63, 64], as also evidenced by their different connectivity with prefrontal and limbic regions. The difference, to some extent, indicates that attention and executive function were preferentially affected by genetically predicted AD in adolescents, followed by dysfunctional emotional information assessment as well as emotional response regulation in adults.

From the whole-brain perspective, the age-specific brain changes explained by AD genetic risk shown in our results are consistent with the classical pattern of damage observed in AD. Significant cortical atrophy was first detected in the cingulate in the early stages, followed by broad areas in the frontal cortex in progression to mild AD [10]. Additionally, a large body of literature supports widespread white matter tract microstructural abnormalities. Notably, the cingulate cingulum, connecting the cingulate cortex and hippocampus, is one of the most severe imaging changes in early AD patients [65]. We, therefore, speculate that AD pathology would partly spread along it from the cingulate cortex to the hippocampus and other brain regions.

Prior studies have noted the importance of different genetic associations with brain development, maturation, and atrophy between childhood and adulthood. For example, the brain-derived neurotrophic factor Val^66^Met carriers have larger right hippocampal volume in children aged 6–10, which is inconsistent with adult findings [66–68]. Margarida et al. also hold the view that during brain development, genes relevant to brain disorders show distinct temporal characteristics [69]. Our results extend this literature by analyzing the correlation of AD PRS with the macro- and microstructure of the brain in two diverse age groups to identify age-specific patterns of brain development and senescence. Future research will need to determine whether these findings reflect distinguishable brain development and aging trajectories and indicate a clear link between spatiotemporal characters and the MRI phenotypic manifestations.

Moreover, the results of the subsequent analysis with the *APOE*-independent PRS have also shown spatial and temporal specificity. The *APOE*-independent PRS is mainly associated with white matter change in adolescence, while the effects of *APOE* predominantly spread to the whole cerebral tissues during middle and older adulthood. These results generally agree with those obtained in previous studies demonstrating that *APOE* has a pattern of brain expression most pronounced during adulthood rather than early development [70]. This *APOE* effect leads to a loss of microstructural organization and thus affects white matter integrity as a function of increasing age [10, 70].

### Strengths and limitations

This investigation is, to our knowledge, the first and largest investigation to examine the associations between AD PRS and whole brain structures at regional scales. Our study strength includes the utilization of two independent datasets with different age ranges, covering adolescence and adulthood to assess the differences. However, there are several limitations we must acknowledge. A major limitation is the predominantly European ancestry participants in our study, which minimized the population stratification bias but limited the generalizability of our findings to other populations [71]. This common problem in such research is primarily due to the lack of large sample size GWAS and cohorts of other ethnicities, and future research should be warranted in other populations to fully understand the biological pathways of AD. Second, the limited age span (9–11 and 46–82 years) of two datasets from adolescents to middle adulthood cannot completely cover the stage of brain development and cognitive decline, thus preventing us from capturing the full trajectory of brain structure changes explained by AD-related genes. Third, given some uncontrollable differences between two datasets (such as image processing methods and sample size), making a direct quantitative comparison is not technically feasible. Future studies with even larger sample sizes, wider age spans, and more diverse MRI metrics are warranted to increase the sensitivity for detecting obvious genetic effects at early stages of brain development and validate such dynamic changes. Fourth, the additional variance that PRS accounted for we found was relatively small (< 1%), which is possibly due to the complex multidimensional properties of the brain.

## Conclusion

By combining two wide age-spanning non-demented populations, the current study discovered evidence that using PRS for AD demonstrated associations with changes in MRI metrics of the macrostructure and microstructure of the brain at various life stages. We also identify age-specific brain change consistent with the classical pattern of brain impairment observed in AD patients. These findings suggest that genomic vulnerability to AD may be partly mediated through brain abnormalities before the significant manifestation of clinical symptoms by several decades and thus may be useful for PRS-based AD risk assessment and pave the way for further prevention trials of AD. Future longitudinal studies are required to confirm such dynamic changes in the brain regions we investigated here and to completely comprehend the relationships between structural and functional brain impairments.

## Supplementary Information

**Additional file 1:** **Supplementary Methods 1.** MRI data Acquisition and Processing in UKB. **SupplementaryMethods 2. **MRI data Acquisition and Processing in ABCD. SupplementaryMethods 3. Included measures. **Fig. S1.** Polygenic risk scores. **Fig.S2.** Associations between AD PRS (constructed using GWAS data reported by Kunkle et al.) and cortical macrostructural MRI metrics in ABCD. **Fig.S3.** Associations between AD PRS (constructed using GWAS data reported by Kunkle et al.) and volume of subcortical structures in ABCD. **Fig.S4.** Associations between AD PRS (constructed using GWAS data reported by Kunkle et al.) and white matter microstructural MRI metrics inABCD. **Fig. S5.** The correlation between PRSs constructed by different GWAS data (Schwartzentruber et al. 2021 and Kunkle et al. 2019) in ABCD sample. **Fig. S6.** Associations between AD PRS (excluding *APOE* along with adjust APOE genotype)and cortical macrostructural MRI metrics. **Fig. S7.** Associations betweenAD PRS (excluding APOE along with adjust APOE genotype) and volume ofsubcortical structures. **Fig. S8.** Associations between AD PRS (excluding *APOE* along with adjust APOE genotype)and white matter microstructural MRI metrics. **Fig. S9.** Trends in thecorrelation between association results (adjust 40PCs or 10PCs) of PRSs withbrain structures in UKB samples. **Fig. S10.** Trends in the correlationbetween association results (adjust birth weight and gestational age or not) ofPRSs with brain structures in ABCD samples. **Fig. S11.** Trends in thecorrelation between association results (outliers simply removed or winsorized)of PRSs with brain structures in ABCD samples. 

**Additional file 2:** **Table S1.** Descriptive details about study sample. **Table S2.** Number of SNPs at each *p*-value inclusion threshold. **Table S3.** A comprehensive list of all MRI metrics examined in our study. **Table S4.** Interaction of PRS-hemisphere for regional cortex (CA, CT, CV). **Table S5.** Interaction of PRS-hemisphere for white matter tracts (FA, MD). **Table S6.** Interaction of PRS-hemisphere for subcortical volume (SV). **Table S7.**Results for association between all eight PRS and regional cortex (CA, CT, CV). **Table S8.** Results for association between all eight PRS and subcortical volume (SV). **Table S9.** Results for association between all eight PRS and whitematter tracts (FA, MD). **Table S10.** Sensitivity analysis results (ABCD -(Kunkle et al. 2019)) for association between all eight PRS and regional cortex (CA, CT, CV). **Table S11.** Sensitivity analysis results (ABCD -(Kunkle et al. 2019)) for association between all eight PRS and subcortical volume(SV). **Table S12.** Sensitivity analysis results (ABCD - (Kunkle et al.2019)) for association between all eight PRS and white matter tracts (FA, MD). **Table S13.** Results for association between all eight PRS (excluding APOE  along with adjust APOE genotype) and regional cortex(CA, CT, CV). **Table S14.** Results for association between all eight PRS (excluding APOE along with adjust APOE genotype) and subcortical volume (SV). **Table S15.** Results for association between all eight PRS (excluding APOE along with adjust APOE genotype) and whitematter tracts (FA, MD). **Table S16.** Sensitivity analysis results (UKB -adjust 40PCs) for association between all eight PRS and regional cortex (CA, CT, CV). **Table S17.** Sensitivity analysis results (UKB - adjust 40PCs) for association between all eight PRS and subcortical volume (SV). **Table S18.**Sensitivity analysis results (UKB - adjust 40PCs) for association between all eight PRS and white matter tracts (FA, MD). **Table S19.** Sensitivity analysis results (ABCD - adjust birth weight and gestational age) for association between all eight PRS and regional cortex (CA, CT, CV). **Table S20.** Sensitivity analysis results (ABCD - adjust birth weight andgestational age) for association between all eight PRS and subcortical volume(SV). **Table S21.** Sensitivity analysis results (ABCD - adjust birthweight and gestational age) for association between all eight PRS and whitematter tracts (FA, MD). **Table S22.** Sensitivity analysis results (outliers winsorized) for association between all eight PRS and regional cortex (CA, CT, CV). **Table S23.** Sensitivity analysis results (outliers winsorized) for association between all eight PRS and subcortical volume (SV). **TableS24.** Sensitivity analysis results (outliers winsorized) for association between all eight PRS and white matter tracts (FA, MD).

## Data Availability

The ABCD data that support the findings of this study are openly available in the ABCD Dataset Data Release 2.01 at https://nda.nih.gov/abcd. The UKB data used in this study were accessed from the publicly available UK Biobank Resource under application number #19,542, which cannot be shared according to European law (General Data Protection Regulation). However, UKB data could be available on request via the UK Biobank (www.ukbiobank.ac.uk).

## Abbreviations

SNPs: Single nucleotide polymorphisms
GWAS: Genome-wide association studies
UKB: UK Biobank
ABCD: Adolescent Brain Cognitive Development Study
IGAP: International Genomics of Alzheimer’s Project
PT: *p*-Value thresholds
CA: Cortical area
CT: Cortical thickness
CV: Cortical volume
SV: Subcortical volume
FDR: False discovery rate
PC: Principal components
SD: Standard deviation
