# Supplementary material for: Association between polygenic risk for Alzheimer’s disease and brain structure in children and adults
Source: Alzheimers Res Ther. 2023 Jun 13;15:109. doi: 10.1186/s13195-023-01256-z (PMC10262429; doi:10.1186/s13195-023-01256-z)
Supplement: Supplementary file 1 — Additional file 1: Supplementary Methods 1. MRI data Acquisition and Processing in UKB. SupplementaryMethods 2. MRI data Acquisition and Processing in ABCD. SupplementaryMethods 3. Included measures. Fig. S1. Polygenic risk scores. Fig.S2. Associations between AD PRS (constructed using GWAS data reported by Kunkle et al.) and cortical macrostructural MRI metrics in ABCD. Fig.S3. Associations between AD PRS (constructed using GWAS data reported by Kunkle et al.) and volume of subcortical structures in ABCD. Fig.S4. Associations between AD PRS (constructed using GWAS data reported by Kunkle et al.) and white matter microstructural MRI metrics inABCD. Fig. S5. The correlation between PRSs constructed by different GWAS data (Schwartzentruber et al. 2021 and Kunkle et al. 2019) in ABCD sample. Fig. S6. Associations between AD PRS (excluding APOE along with adjust APOE genotype)and cortical macrostructural MRI metrics. Fig. S7. Associations betweenAD PRS (excluding APOE along with adjust APOE genotype) and volume ofsubcortical structures. Fig. S8. Associations between AD PRS (excluding APOE along with adjust APOE genotype)and white matter microstructural MRI metrics. Fig. S9. Trends in thecorrelation between association results (adjust 40PCs or 10PCs) of PRSs withbrain structures in UKB samples. Fig. S10. Trends in the correlationbetween association results (adjust birth weight and gestational age or not) ofPRSs with brain structures in ABCD samples. Fig. S11. Trends in thecorrelation between association results (outliers simply removed or winsorized)of PRSs with brain structures in ABCD samples. [file 13195_2023_1256_MOESM1_ESM.docx]

**Supplementary material Content**

**Association between polygenic risk for Alzheimer’s disease and brain structure in children and adults**

[Supplementary Methods 1 MRI data Acquisition and Processing in UKB 1](#_Toc130855076)

[Supplementary Methods 2 MRI data Acquisition and Processing in ABCD 2](#_Toc130855077)

[Supplementary Methods 3 Included measures 4](#_Toc130855078)

[Figure S1. Polygenic risk scores. 4](#_Toc130855079)

[Figure S2. Associations between AD PRS (constructed using GWAS data reported by Kunkle et al.) and cortical macrostructural MRI metrics in ABCD 6](#_Toc130855080)

[Figure S3. Associations between AD PRS (constructed using GWAS data reported by Kunkle et al.) and volume of subcortical structures in ABCD 7](#_Toc130855081)

[Figure S4. Associations between AD PRS (constructed using GWAS data reported by Kunkle et al.) and white matter microstructural MRI metrics in ABCD 7](#_Toc130855082)

[Figure S5. The correlation between PRSs constructed by different GWAS data (Schwartzentruber et al. 2021 and Kunkle et al. 2019) in ABCD sample 8](#_Toc130855083)

[Figure S6. Associations between AD PRS (excluding *APOE* along with adjust APOE genotype) and cortical macrostructural MRI metrics 9](#_Toc130855084)

[Figure S7. Associations between AD PRS (excluding *APOE* along with adjust APOE genotype) and volume of subcortical structures 10](#_Toc130855085)

[Figure S8. Associations between AD PRS (excluding *APOE* along with adjust APOE genotype) and white matter microstructural MRI metrics 11](#_Toc130855086)

[Figure S9. Trends in the correlation between association results (adjust 40PCs or 10PCs) of PRSs with brain structures in UKB samples 12](#_Toc130855087)

[Figure S10. Trends in the correlation between association results (adjust birth weight and gestational age or not) of PRSs with brain structures in ABCD samples 12](#_Toc130855088)

[Figure S11. Trends in the correlation between association results (outliers simply removed or winsorized) of PRSs with brain structures in ABCD samples 13](#_Toc130855089)

# Supplementary Methods 1 MRI data Acquisition and Processing in UKB

**Structural Imaging** The full detail of the MRI data acquisition protocol and pipeline for the generation of imaging derived phenotypes (IDPs) can be found in the open-access articles [1, 2]. Briefly, Neuroimaging MRI data with a resolution of 1 x 1 x 1 mm were collected using a standard Siemens Skyra 3T (Siemens Healthcare, Erlangen, Germany) scanner with a 32-channel head coil. Then T1-weighted images were obtained with a 3D magnetization-prepared rapid gradient echo (MPRAGE) sequence as the scanning protocol.

UK Biobank team processed structural imaging and made it available for researchers from UK Biobank upon data access application. FreeSurfer was used to analyze T1 images, using surface templates (Desikan-Killiany parcellation) to obtain IDPs available in UKB category of FreeSurfer aparc (ID=192), referring to the cortical area, cortical volume, and mean cortical thickness of 34 bilateral cortical regions. FSL’s FIRST was implemented to extract volume of 7 bilateral key subcortical structures, including accumbens, amygdala, thalamus, hippocampus, pallidum, caudate, putamen [3], corresponding to UKB category ID 1102.

**Diffusion Magnetic Resonance Imaging** Diffusion-weighted MRI data comprised a spin-echo echo-planar imaging sequence with 100 distinct diffusion-encoding directions and 2 mm isotropic voxels, a 104 x 104 mm field of view, and a multiband acceleration factor of 3 [1]; EPI distortions and eddy currents/outlier slices were respectively addressed with FSl’s topup and eddy. artefacts introduced by head motion were minimized with gradient distortion correction. Then the diffusion-weighted images were submitted to a tractography-based analysis including BEDPOSTX (Bayesian Estimation of Diffusion Parameters Obtained using Sampling Techniques) and PROBTRACKX, followed by mapping 27 major tracts using the AutoPtx package from FSL. After these preprocessing steps, the quality-controlled IDPs including the weighted-mean fractional anisotropy (FA) and mean diffusion (MD) for each of the 27 tracts were generated.

# Supplementary Methods 2 MRI data Acquisition and Processing in ABCD

Our investigation used the data from the Adolescent Brain Cognitive Development Study (ABCD) consortium annual curated data release 3.0, also including a subset of brain scan data for the second timepoint. 11 556 of the 11 878 participants in the ABCD had structural MRI data. The imaging data acquired from ABCD release 2.0.1 corrected issues with data release 2.0 with regarding data laterality in some participants. Previous researches [4, 5] have provided a detailed description of standardized imaging acquisition parameters and protocol, and image preprocessing and analysis methodology in the ABCD study. The neuroimaging data acquisition and analysis procedures used by the ABCD study that are pertinent to our main analyses are briefly outlined below.

**Structural Imaging** To obtain 1 mm isotropic T1-weighted structural images, 3T MRI scanners (Siemens, Phillips and GE) with either a 32-channel head or 64-channel head-and-neck coil were employed. MRI scan protocols were meticulously synchronized between the three MRI vendor platforms to reduce variation across scanners. Real-time motion detection and correction were implemented to mitigate head motion in pediatric populations. Furthermore, prospective motion correction was implemented in GE and Volumetric Navigators (vNav) were used in the Siemens the Philips platforms during the structural data acquisition.

MRI data was processed using the Multi-Modal Processing Stream software package, including FreeSurfer 5.3, for cortical and subcortical reconstruction and volumetric parcellation (available at <http://surfer.nmr.mgh.harvard.edu/>). Only those whose structural MRI reconstructions passed QC tests (n=11 076) were retained, as described in detail by Hagler et al. [4]. The cortical [6] and subcortical [7] structures were labeled with atlas-based segmentation. The ABCD standard pipeline specifies a modified intensity normalization process using both continuity and intensity information, to generate structural phenotypes such as cortical thickness, cortical area, and cortical volume.

**Diffusion Magnetic Resonance Imaging** The diffusion tensor imaging (DTI) data were obtained by using a multiband EPI sequence and high angular resolution diffusion imaging scans, with slice acceleration factor 3 and 96 diffusion directions. Each DTI acquisition data included fieldmap scans for correction of spatial and intensity distortion caused by B_0_ field inhomogeneity, using an accurate procedure that relies on reversing gradient method. Eddy current correction (ECC) for diffusion data used an iterative model-based approach, predicting the pattern of distortions across the entire set of diffusion-weighted volumes in terms of translation, scaling, and limited to shear along the phase-encode direction based on diffusion gradient orientations and amplitudes [8]. Rigid body registration of each data frame with the corresponding eddy current-corrected volume was used to correct additional subtle types of head motion. To ensure precise model fitting and tractography, the diffusion gradient matrix was adjusted for head rotation.

AtlasTrack, a probabilistic atlas-based method, were used to label major white matter tracts. Voxels identified by FreeSurfer's automated brain segmentation to contain primarily gray matter or cerebral spinal fluid were excluded from analysis. After fitting the diffusion tensor, several standard microstructural tissue measures, including fractional anisotropy (FA) and mean diffusivity (MD), were generated using a standard linear estimation approach with log-transformed diffusion-weighted signals.

# Supplementary Methods 3 Included measures

The cortical gray matter measures included for PRS analysis were these 3 parameters: cortical thickness, cortical area and cortical volume of 34 bilateral vertex regions aligned to the Desikan atlas. For white matter modalities, we used FA and MD, which are the most widely used in neuroimaging research (5). And we chose 10 major white matter tracts across left and right hemispheres in our main analysis for methodological consistency because they were available in both ABCD and UKB datasets. As for the subcortical measures, we chose volumetric metric of 7 bilateral key subcortical structures.

For a comprehensive list of all MRI metrics examined in our study, please see Table S1.

# Figure S1. Polygenic risk scores.


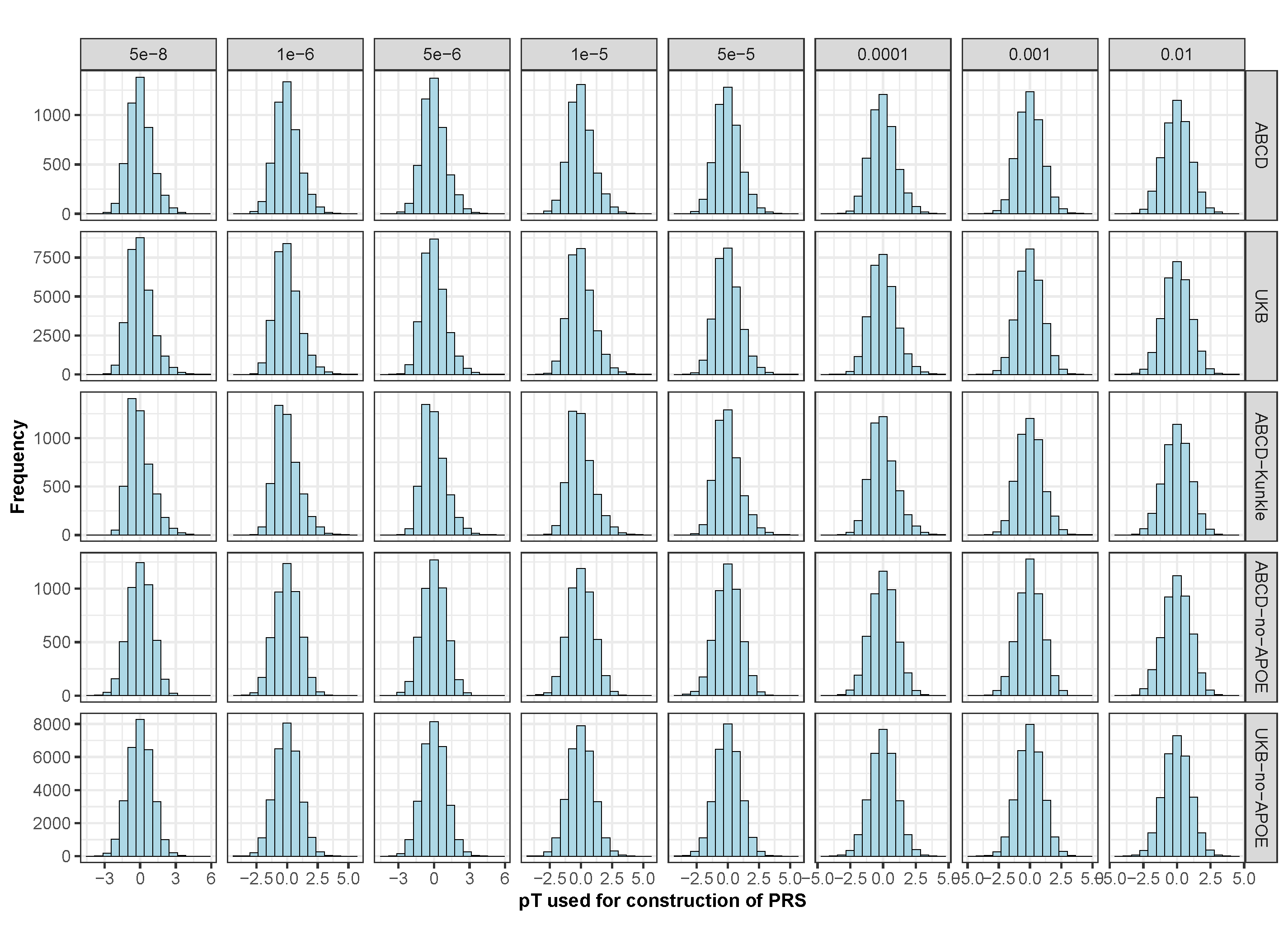


Frequency histograms of scaled polygenic risk scores (PRS) of Alzheimer’s disease at all eight p value thresholds, which satisfy the normal distribution within both ABCD and UKB samples.

# Figure S2. Associations between AD PRS (constructed using GWAS data reported by Kunkle et al.) and cortical macrostructural MRI metrics in ABCD


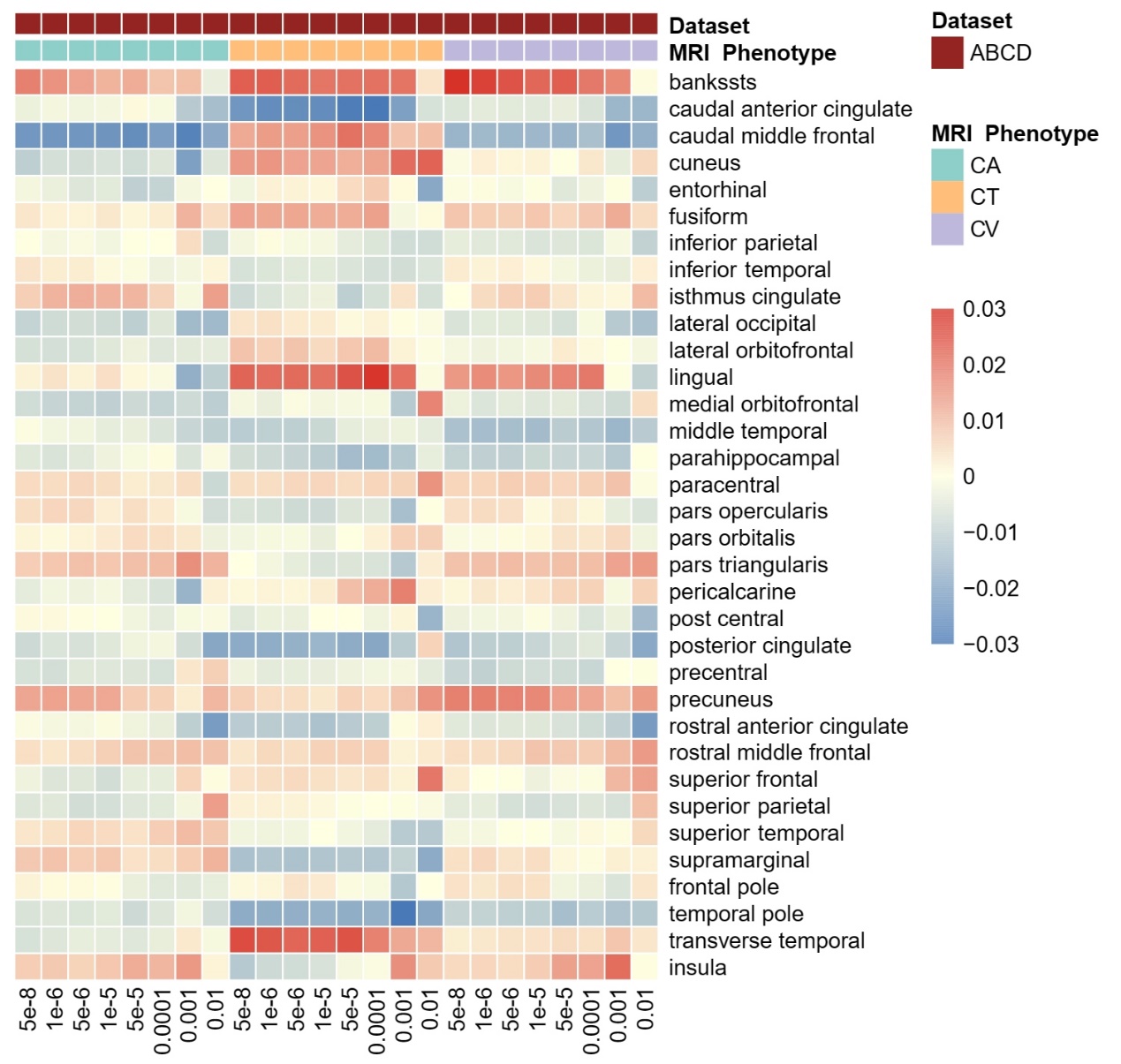


Heatmap of regional associations between cortical CA, CT, CT and all eight AD PRS (constructed using GWAS data reported by Kunkle et al.); The rows represent the 34 cortical regions and the columns represent the eight AD PRSs used in our study.

# Figure S3. Associations between AD PRS (constructed using GWAS data reported by Kunkle et al.) and volume of subcortical structures in ABCD

Barcharts of variance explained by AD PRS (R^2^, y-axis) constructed at each of eight p value thresholds (x-axis) for volume of 7 subcortical structures: accumbens, amygdala, thalamus, hippocampus, pallidum, caudate, putamen. Red and blue colors respectively correspond to positive and negative associations.


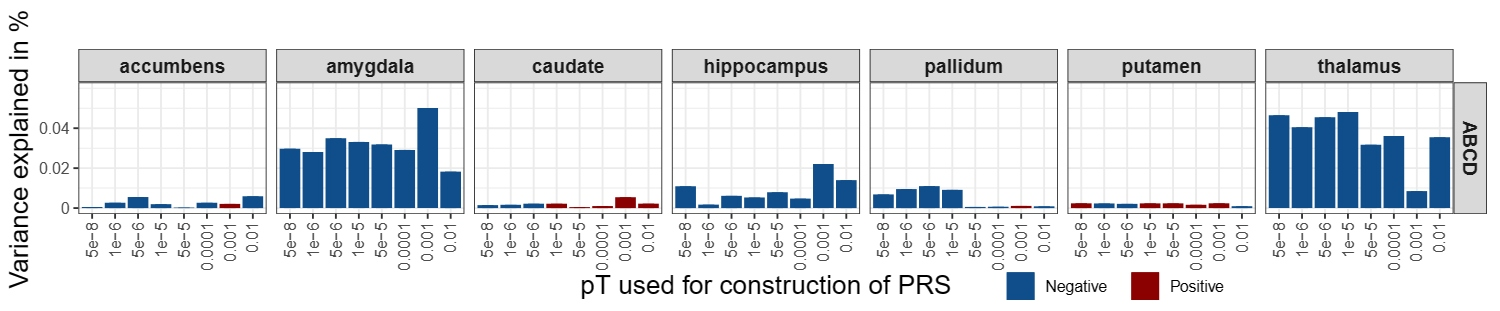


# Figure S4. Associations between AD PRS (constructed using GWAS data reported by Kunkle et al.) and white matter microstructural MRI metrics in ABCD

Barcharts of variance explained by AD PRS (R^2^, y-axis) constructed at each of eight p value thresholds (x-axis) for two white matter metrics (FA, fractional anisotropy; MD, mean diffusivity) measured at 10 major white matter tracts: ifo, inferior fronto-occipital fasciculus; ilf, inferior longitudinal fasciculus; slf, superior longitudinal fasciculus; unc, uncinate fasciculus; fma, forceps major; fmi, forceps minor; cgc, cingulate gyrus part of cingulum; cgh, parahippocampal part of cingulum; atr, anterior thalamic radiation; cst, corticospinal tract.

Red and blue colors respectively correspond to positive and negative associations.


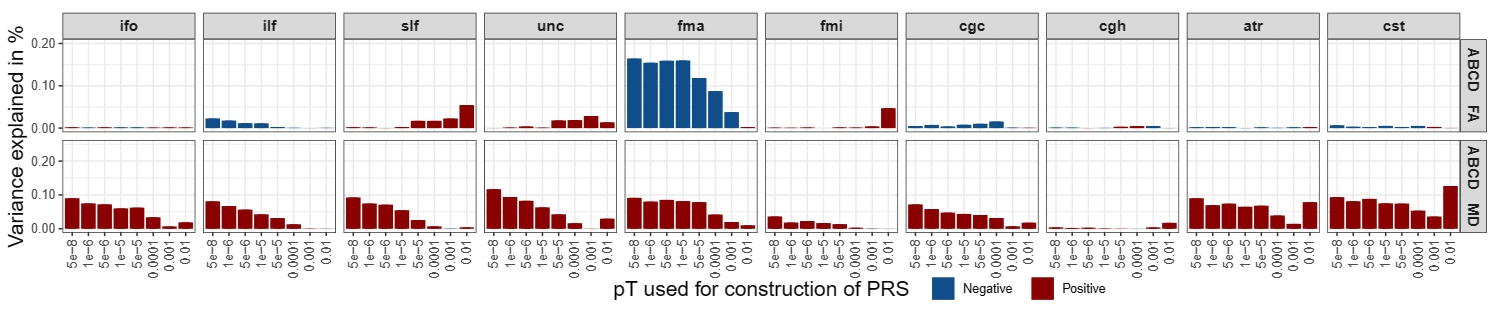


# Figure S5. The correlation between PRSs constructed by different GWAS data (Schwartzentruber et al. 2021 and Kunkle et al. 2019) in ABCD sample

Linear trendlines were established and the Pearson correlation coefficient (R) and associated p value are shown. The horizontal axis represents PRS constructed using AD GWAS reported by Schwartzentruber et al., and the vertical axis represents PRS constructed using AD GWAS reported by Kunkle et al.

**A** Trends in the correlation between PRSs constructed by different GWAS data (Schwartzentruber et al. 2021 and Kunkle et al. 2019) in ABCD sample at different PTs.

**B, C** Trends in the correlation between different PRSs and brain structure association results (beta and -log_10_p) in ABCD sample

**
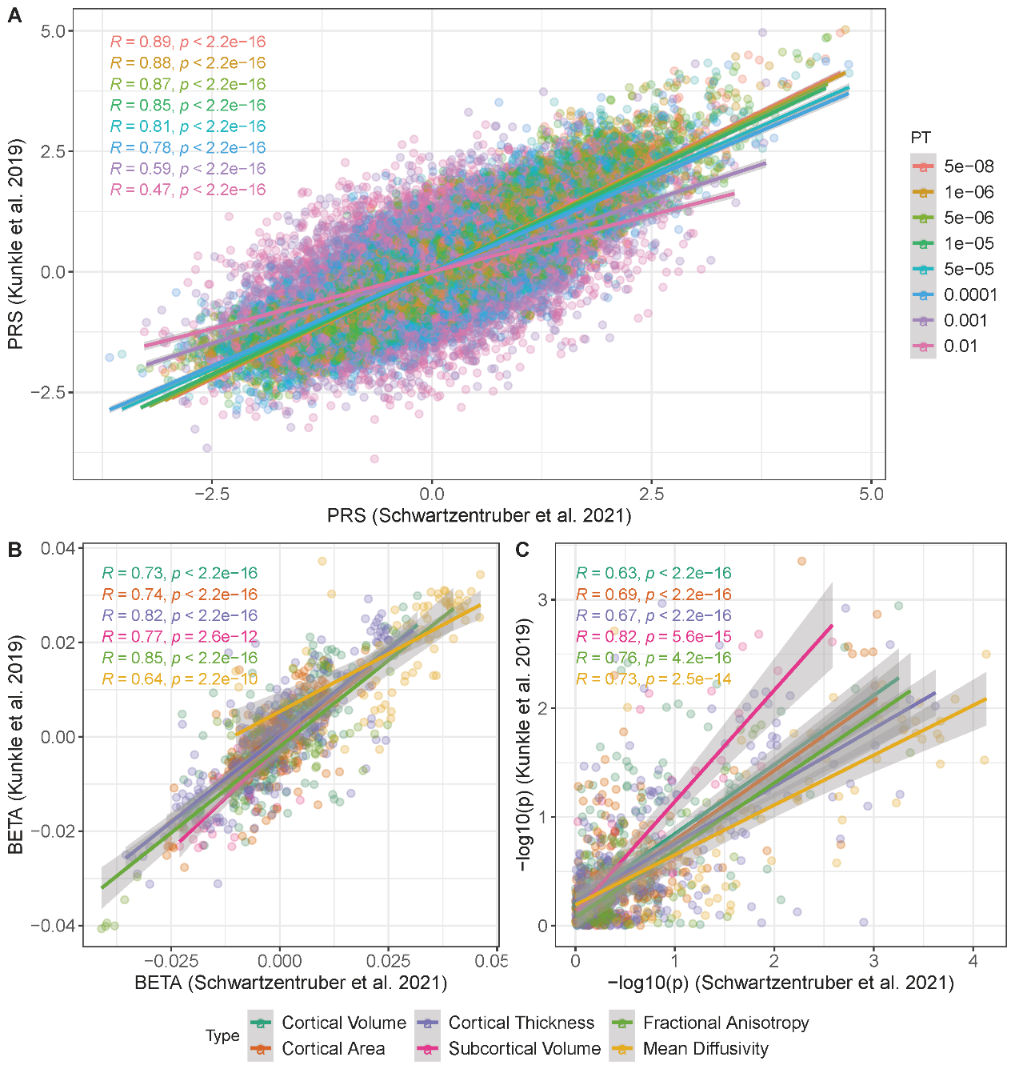
**

# Figure S6. Associations between AD PRS (excluding *APOE* along with adjust APOE genotype) and cortical macrostructural MRI metrics

Heatmap of regional associations between cortical CA, CT, CV and all eight AD PRS；The rows represent the 34 cortical regions and the columns represent the eight AD PRSs used in our study. Asterisks indicate P values after FDR correction: *FDR < 0.05, **FDR < 0.01.


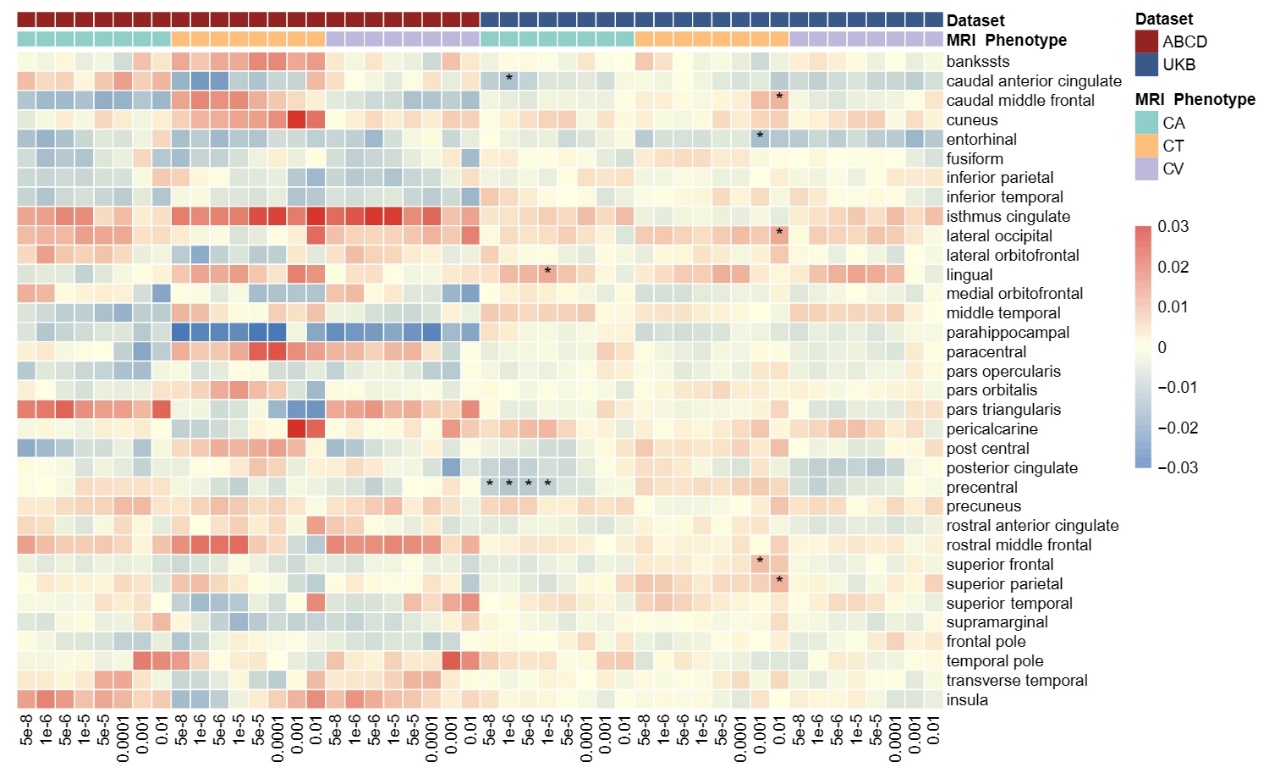


# Figure S7. Associations between AD PRS (excluding *APOE* along with adjust APOE genotype) and volume of subcortical structures

Barcharts of variance explained by AD PRS (R2, y-axis) constructed at each of eight p value thresholds (x-axis) for volume of 7 subcortical structures: accumbens, amygdala, thalamus, hippocampus, pallidum, caudate, putamen. Red and blue colors respectively correspond to positive and negative associations. Asterisks indicate P values after FDR correction: *FDR < 0.05, **FDR < 0.01, ***FDR < 0.001.


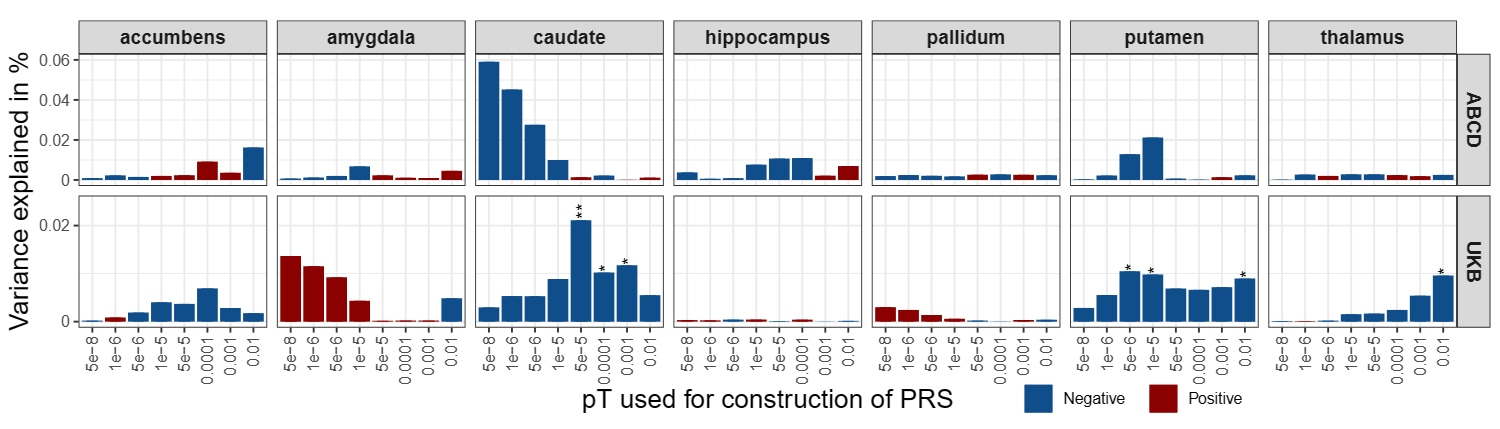


# Figure S8. Associations between AD PRS (excluding *APOE* along with adjust APOE genotype) and white matter microstructural MRI metrics

Barcharts of variance explained by AD PRS (R2, y-axis) constructed at each of eight p value thresholds (x-axis) for two white matter metrics (FA, fractional anisotropy; MD, mean diffusivity) measured at 10 major white matter tracts: ifo, inferior fronto-occipital fasciculus; ilf, inferior longitudinal fasciculus; slf, superior longitudinal fasciculus; unc, uncinate fasciculus; fma, forceps major; fmi, forceps minor; cgc, cingulate gyrus part of cingulum; cgh, parahippocampal part of cingulum; atr, anterior thalamic radiation; cst, corticospinal tract.

Red and blue colors respectively correspond to positive and negative associations. Asterisks indicate P values after FDR correction: *FDR < 0.05, **FDR < 0.01.


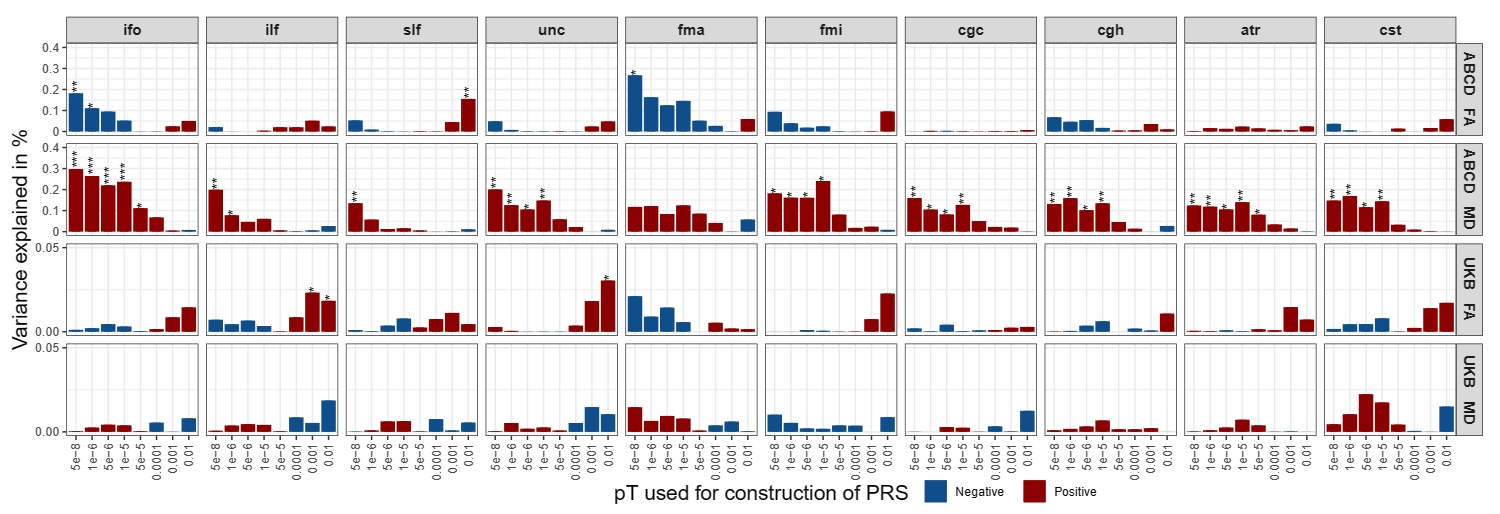


# Figure S9. Trends in the correlation between association results (adjust 40PCs or 10PCs) of PRSs with brain structures in UKB samples

Linear trendlines were established and the Pearson correlation coefficient (R) and associated p value are shown. The horizontal axis represents the results adjust 10PCs, and the vertical axis represents the results adjust 40PCs

**
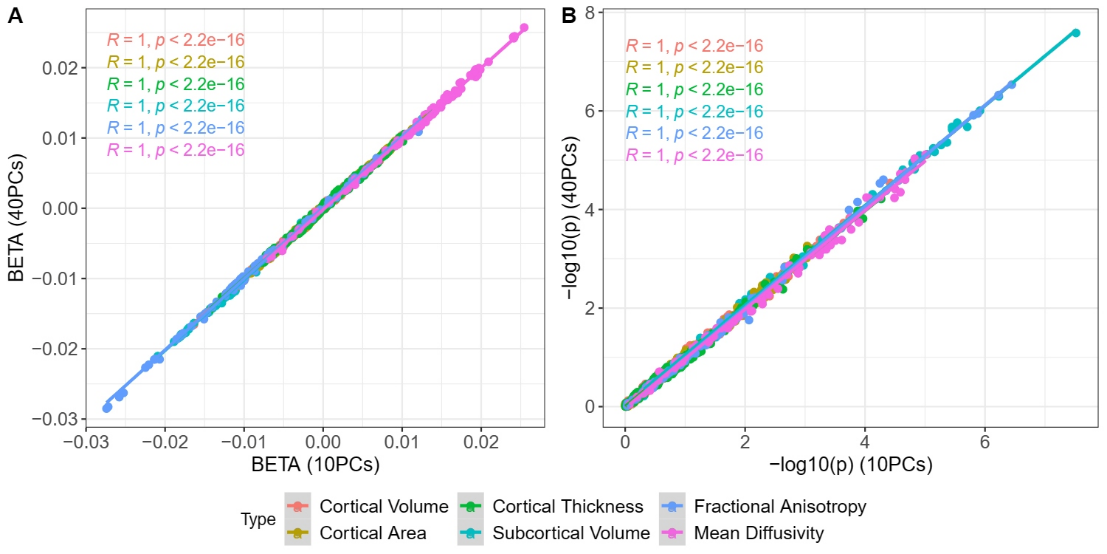
**

# Figure S10. Trends in the correlation between association results (adjust birth weight and gestational age or not) of PRSs with brain structures in ABCD samples

Linear trendlines were established and the Pearson correlation coefficient (R) and associated p value are shown. The horizontal axis represents the results not adjust birth weight and gestational age, and the vertical axis represents the results adjust birth weight and gestational age.

**
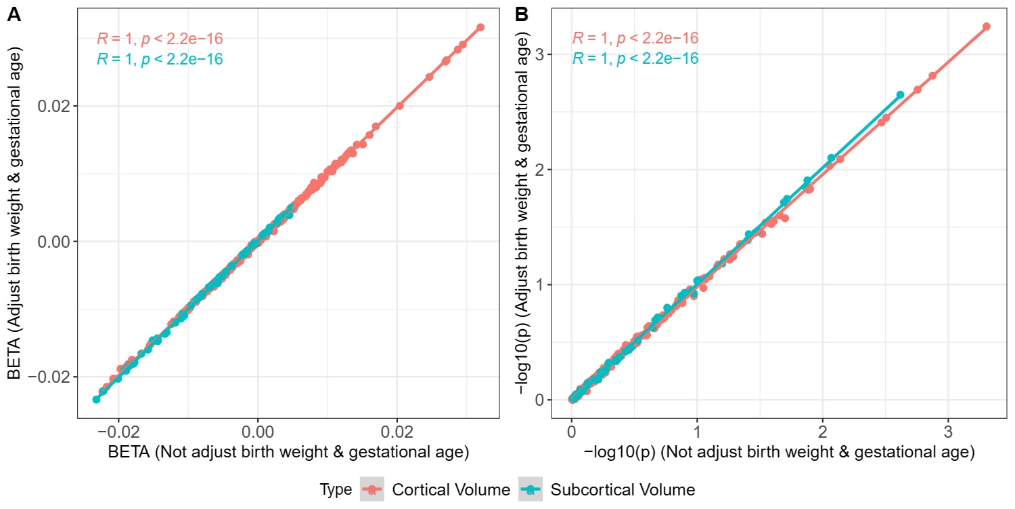
**

# Figure S11. Trends in the correlation between association results (outliers simply removed or winsorized) of PRSs with brain structures in ABCD samples

Linear trendlines were established and the Pearson correlation coefficient (R) and associated p value are shown. The horizontal axis represents the results with outliers simply removed, and the vertical axis represents the results with outliers winsorized.

**AB** for UKB; **CD** for ABCD;

**
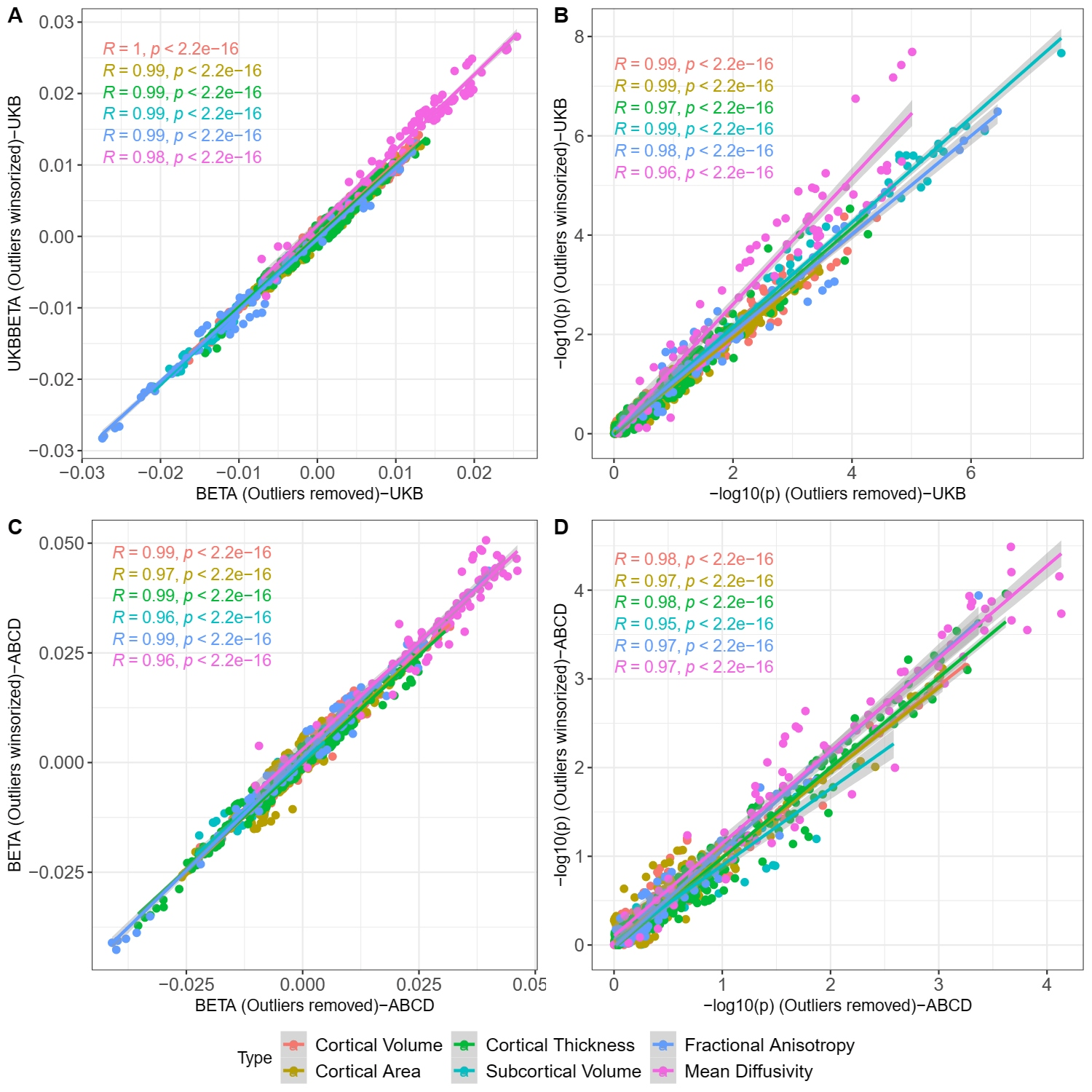
**

**Reference**

1. Miller KL, Alfaro-Almagro F, Bangerter NK, Thomas DL, Yacoub E, Xu J, et al. Multimodal population brain imaging in the UK Biobank prospective epidemiological study. Nat Neurosci. 2016;19:1523-1536.

2. Alfaro-Almagro F, Jenkinson M, Bangerter NK, Andersson JLR, Griffanti L, Douaud G, et al. Image processing and Quality Control for the first 10,000 brain imaging datasets from UK Biobank. Neuroimage. 2018;166:400-424.

3. Patenaude B, Smith SM, Kennedy DN, Jenkinson M. A Bayesian model of shape and appearance for subcortical brain segmentation. Neuroimage. 2011;56:907-922.

4. Hagler DJ, Jr., Hatton S, Cornejo MD, Makowski C, Fair DA, Dick AS, et al. Image processing and analysis methods for the Adolescent Brain Cognitive Development Study. Neuroimage. 2019;202:116091.

5. Casey BJ, Cannonier T, Conley MI, Cohen AO, Barch DM, Heitzeg MM, et al. The Adolescent Brain Cognitive Development (ABCD) study: Imaging acquisition across 21 sites. Dev Cogn Neurosci. 2018;32:43-54.

6. Desikan RS, Ségonne F, Fischl B, Quinn BT, Dickerson BC, Blacker D, et al. An automated labeling system for subdividing the human cerebral cortex on MRI scans into gyral based regions of interest. Neuroimage. 2006;31:968-980.

7. Fischl B, Salat DH, Busa E, Albert M, Dieterich M, Haselgrove C, et al. Whole brain segmentation: automated labeling of neuroanatomical structures in the human brain. Neuron. 2002;33:341-355.

8. Zhuang J, Hrabe J, Kangarlu A, Xu D, Bansal R, Branch CA, et al. Correction of eddy-current distortions in diffusion tensor images using the known directions and strengths of diffusion gradients. J Magn Reson Imaging. 2006;24:1188-1193.
